# LIFE BEEF CARBON: a common framework for quantifying grass and corn based beef farms’ carbon footprints

**DOI:** 10.1017/S1751731119002519

**Published:** 2019-10-31

**Authors:** D. O’Brien, J. Herron, J. Andurand, S. Caré, P. Martinez, L. Migliorati, M. Moro, G. Pirlo, J-B Dollé

**Affiliations:** 1 Environment, Soils and Land Use Department, Teagasc, Johnstown Castle, Wexford, Leinster, Ireland; 2 Livestock Systems Department, Animal and Grassland Research and Innovation Centre, Teagasc, Moorepark, Fermoy, Co. Cork, Ireland; 3 Environment Department, Idele, Site de Monvoisin, 35652 Le Rheu, France; 4 Council for Agricultural Research and Economics – Research Centre for Animal Production and Aquaculture (CREA), via Antonio Lombardo 11, 26900 Lodi, Italy; 5 ASOPROVAC, Calle Orense 6, Madrid, Spain; 6 Environment Head Department, Idele, 54-56 avenue Roger Salengro, 62051 St Laurent Blangy, France

**Keywords:** cattle, greenhouse gas, sustainability, modelling, life cycle assessment

## Abstract

Europe’s roadmap to a low-carbon economy aims to cut greenhouse gas (**GHG**) emissions 80% below 1990 levels by 2050. Beef production is an important source of GHG emissions and is expected to increase as the world population grows. LIFE BEEF CARBON is a voluntary European initiative that aims to reduce GHG emissions per unit of beef (carbon footprint) by 15% over a 10-year period on 2172 farms in four large beef-producing countries. Changes in farms beef carbon footprint are normally estimated via simulation modelling, but the methods current models apply differ. Thus, our initial goal was to develop a common modelling framework to estimate beef farms carbon footprint. The framework was developed for a diverse set of Western Europe farms located in Ireland, Spain, Italy and France. Whole farm and life cycle assessment (**LCA**) models were selected to quantify emissions for the different production contexts and harmonized. Carbon Audit was chosen for Ireland, Bovid-CO_2_ for Spain and CAP’2ER for France and Italy. All models were tested using 20 case study farms, that is, 5 per country and quantified GHG emissions associated with on-farm live weight gain. The comparison showed the ranking of beef systems gross carbon footprint was consistent across the three models. Suckler to weaning or store systems generally had the highest carbon footprint followed by suckler to beef systems and fattening beef systems. When applied to the same farm, Carbon Audit’s footprint estimates were slightly lower than CAP’2ER, but marginally higher than Bovid-CO_2_. These differences occurred because the models were adapted to a specific region’s production circumstances, which meant their emission factors for key sources; that is, methane from enteric fermentation and GHG emissions from concentrates were less accurate when used outside their target region. Thus, for the common modelling framework, region-specific LCA models were chosen to estimate beef carbon footprints instead of a single generic model. Additionally, the Carbon Audit and Bovid-CO_2_ models were updated to include carbon removal by soil and other environmental metrics included in CAP’2ER, for example, acidification. This allows all models to assess the effect carbon mitigation strategies have on other potential pollutants. Several options were identified to reduce beef farms carbon footprint, for example, improving genetic merit. These options were assessed for beef systems, and a mitigation plan was created by each nation. The cumulative mitigation effect of the LIFE BEEF CARBON plan was estimated to exceed the projects reduction target (−15%).

## Implications

Europe’s beef producers are under pressure to reduce greenhouse gas emissions per unit of output, commonly known as carbon footprint. Many models can quantify reductions in beef farms carbon footprint, but they use different approaches. We compared the most widely used models following harmonization of methodology and showed that for a common Western European framework region-specific holistic models were the best way of quantifying beef farms carbon footprints. This finding has important implications for international greenhouse gas assessments and suggests the effects of beef carbon mitigation practices may be under- or overestimated when regional differences are not considered.

## Introduction

Beef is a key product from European Union (**EU**) bovine farming and originates from either the suckler beef herd or dairy herd. Globally, the EU is the third-largest beef producer and typically supplies an annual beef carcass weight (**CW**) equivalent of 7.5 million tonnes (Eurostat, 2017). France is the primary producer of beef in Europe followed by Germany and the UK. Ireland is the region’s largest net exporter of beef, shipping over 90% of its production (Bord Bia, 2016). Italy and Spain both import cattle from Ireland and France, and are the third- and fifth-biggest producers of European beef, respectively.

Beef production has several positive effects on the environment, including protecting grassland ecosystems and conserving soil carbon. This food product, however, like any food, generates undesirable emissions during the production stage. The unwanted emissions associated with food can harm the water, air, soil or wildlife of local ecosystems. Furthermore, some of the emissions from food production, known as greenhouse gas (**GHG**) or carbon emissions, can cause climate change and thus affect global ecosystems. The livestock sector is estimated to be responsible for 14.5% of global GHG emissions, and beef production is the sector’s largest source (Opio *et al.*, 2013). It is expected that EU beef producers will have to minimize GHG emissions as member states pledged to substantially reduce their climate impact at the 21st conference of parties meeting of the United Nations Framework Convention on Climate Change in Paris.

Most GHG emissions from EU beef production are from the non-emission trading sector (**non-ETS**). One of the Europe’s 2030 targets for this sector is to reduce GHG emissions by 30% compared to 2005 levels (European Commission, 2018). The non-ETS 2030 reduction targets for key beef production nations, namely France, Ireland, Italy and Spain, range from 26% to 37%. Achieving these national GHG targets will be challenging given that beef supply needs to increase to feed a growing world population. Global demand for beef is forecasted to grow by 1.2% per annum until 2050 (Opio *et al.*, 2013) and is likely to be partly met by EU producers. The EU beef sector thus needs to measure and cut GHG emissions per unit of output (i.e., carbon footprint or GHG emission intensity).

Measuring annual GHG emissions from livestock sources at the farm or higher scales is normally too difficult. Farm and supply-chain GHG emissions are modelled using equations derived from livestock on experimental farms. The main methods to model GHG emissions associated with beef are life cycle assessment (**LCA**) and whole farm models (Crosson *et al.*, 2011; de Vries *et al.*, 2015). Since 2000, LCA and whole farm models have been developed throughout Europe and North America to quantify beef farming emissions (de Vries *et al.*, 2015). Generally, these models compute emissions that occur prior to cattle leaving the farm for processing. This phase of beef production, known as the cradle to farm-gate phase, is the largest source of GHG emissions and is responsible for about 80% of the GHG emissions associated with beef consumption (Asem-Hiablie *et al.*, 2018).

Whole farm and LCA models have been used to estimate GHG from several EU beef production systems, including Irish grazing systems (Foley *et al.*, 2011; Crosson *et al.*, 2013), French mixed cropping and grass-based farms (Dollé *et al.*, 2011) and Italian and Spanish finishing or feedlot systems (Ridha, 2013; Boselli, 2015). Previous results from these and other LCA models have recommended different measures to reduce GHG emissions. This can be explained in part by the variability in local conditions, for example, soil type, topography and climate, but can be due to inconsistent methodological decisions. Furthermore, some beef LCA models consider other environmental impacts before recommending a GHG mitigation strategy. A recent review by McClelland *et al.* (2018), however, found that 56% of the 173 livestock LCA studies published between 2000 and 2016 investigated three or fewer impact categories. Within these studies the most common impacts investigated were climate change (98% of studies), resource depletion (54% of studies), eutrophication (50% of studies) and acidification (47% of studies), while other important environmental measures such as biodiversity were rarely assessed. This shortcoming of livestock LCA models could inadvertently lead to GHG mitigation measures that have a negative impact on overall environmental performance.

The objective of this study, part of the LIFE BEEF CARBON project, was twofold. First, create a common framework to quantify beef carbon footprints and other important environmental measures. Second, evaluate the framework in terms of beef carbon footprint using farm-level models. Once created, the common framework will be used to evaluate carbon footprints from 2172 commercial beef system across the four countries and applied to assess partner nations’ beef carbon footprint mitigation action plans. This plan will be synthesized used a shared partnership approach.

## Material and methods

### Selection of beef modelling tools

The initial step in developing a common beef framework for GHG emissions and other environmental measure was to conduct an inventory analysis of countries models. Briefly, the analysis reviewed national and European literature regarding beef carbon footprint models and evaluated models’ goals, methods, scope, GHG emission factors and environmental impacts. Beef carbon footprints published by these models were also validated by comparing them to European and global results Opio *et al.* (2013) estimated using the United Nations Food and Agriculture Organization (**FAO**) global livestock environmental assessment (**GLEAM**) model. Any models that did not use a whole-farm/LCA approach or estimate footprints within the normal range were removed. Models were selected from the remaining options based on the type and number of farms they can analyse, and their temporal and geographical relevance.

The inventory analysis showed that 8 of the 13 studies used a cradle to farm-gate LCA model to quantify GHG emissions (Supplementary Material Table S1). The other approach applied was whole farm modelling. Cradle to farm-gate LCA and whole-farm models quantified farms’ annual gross GHG emissions. The gross farm GHG emission encompassed on-farm emissions from animal husbandry and feed production activities. It also included off-farm GHG emissions associated with the production and transport of imported inputs, for example, synthetic mineral fertilizer. Three studies accounted for carbon removal by sinks, for example, grassland soil, which was used to estimate farms’ net GHG emissions, that is, gross farm GHG emissions’ less carbon sequestration. Consistent with GLEAM, most models quantified beef farms gross carbon footprint by relating farms’ gross GHG emission in CO_2_ equivalents (**CO**_**2**_**e**) to live weight (**LW**), live weight gain (**LWG**) and/or CW. To enable a comparison with GLEAM, LW estimates were converted to CW using a 55% dressing percentage. Net beef farm carbon footprint was estimated by relating net GHG emissions to LW production.

Generally, models estimated GHG emissions from a representative farm or research farm. The results for these farms were similar or less than the average GLEAM estimated for European suckler systems in 2005 (32 kg CO_2_e/kg CW). Models applied to estimate GHG emissions from commercial farms reported a greater range in beef carbon footprints than models used for research farms. In addition, the former estimated other environmental impacts at farm level, but most were limited to three or fewer environmental impacts, except an application of the French modelling tool CAP’2ER (Boselli, 2015). The inventory analysis also showed the French model along with Carbon Audit was capable of estimating GHG emissions from a significant number of commercial farms (i.e., 50 or more).

This is a key requirement for LIFE BEEF CARBON and was one of the primary reasons these models were selected from the options available. The latter LCA model was chosen for Ireland because it is ubiquitously used and adapted to local conditions. The CAP’2ER LCA model was chosen for French farms for similar reasons. Although tools are available to quantify the main environmental categories of Italian beef systems (e.g., Berton *et al.*, 2016), CAP’2ER was chosen for Italy in order to use a consistent approach to link commercial French suckler to weaning systems to Italian finishing systems (Gac and Boselli, 2014). Furthermore, research by Boselli *et al.* (2015) and Berton *et al.* (2017) demonstrated it was suitable to use for common commercial Italian beef systems. Neither CAP’2ER nor Carbon Audit models are adapted to Spanish beef farming systems. However, the Spanish models we assessed were not capable of estimating GHG emissions at a large scale. Thus, an alternative farm-level LCA model, Bovid-CO_2_, was developed and selected for Spain.

### Farm-level modelling tools

Carbon Audit, CAP’2ER and Bovid-CO_2_ quantify the cradle to farm-gate carbon footprint of beef. The farm-level models required the same key inputs to estimate GHG emissions from beef production, for example, cropland area, grassland area, cattle inventories, cattle purchase and sale weights, N fertilizer and lime application, concentrate feed, housing and grazing periods, manure storage systems, manure application profiles and fossil fuel consumption. Across models, data for minor inputs (e.g., electricity) were based on literature values if no data were available. The gross carbon footprints of beef farms, that is, GHG emission/unit of output, were estimated by models using various metrics namely CW, LW or LWG. The latter was chosen to compare model footprints. Cattle LW was normally only produced on beef farms; thus, all GHG emissions were allocated to this output.

The CAP’2ER model can estimate the net carbon footprint (gross footprint less carbon sequestration) and relate other environmental impacts to LWG, that is, acidification, eutrophication, non-renewable energy use and biodiversity. The models’ methods are described by Idele (2018). However, Carbon Audit and Bovid-CO_2_ were originally developed only to quantify GHG emissions. Thus, the comparison was confined to carbon footprint. Supplementary Material Table S2 lists the on-farm and off-farm GHG sources and sinks considered and details each model’s key emission factors. The on-farm sources of GHG emissions estimated were methane (CH_4_) from cattle feed digestion (enteric fermentation), CH_4_ and nitrous oxide (N_2_O) from manure, CO_2_ and N_2_O from fertilizer application and indirect N_2_O emissions from ammonia (NH_3_) and nitrate re-deposition. Off-farm sources of GHG emissions included fertilizer manufacture, concentrate production, electricity generation and other inputs, for example, limestone.

Most tools calculations for on-farm GHG sources were from the Intergovernmental Panel on Climate Change (**IPCC**) guidelines and national GHG inventory reports. The emission factors for off-farm sources were usually only available in CO_2_e and obtained from external databases, for example, Carbon Trust (2013). A 100-year time horizon was assumed by all models when converting GHG emissions to CO_2_e, but different global warming potential factors were used. The factors were standardized across tools to 25 for CH_4_, 298 for N_2_O and 1 for CO_2_ (IPCC, 2007). Carbon sequestration by grassland and hedgerows is included in CAP’2ER using the research findings of Dollé *et al.* (2013). Carbon audit and Bovid-CO_2_ were revised to include this sink.

### Application of beef carbon footprint models

The models were applied to quantify the GHG emissions from 20 case study beef cattle farms, that is, five farms per country. The five Irish case studies were based on research farm-lets reported by Clarke *et al.* (2013) and Murphy *et al.* (2017). These farms generally fed low levels of concentrate and were dependent on grass. The Irish farms grazed cattle during the spring, summer and autumn. Two of the five Irish case studies operated suckler or beef cow systems, that is, suckler to weaning and suckler to beef. The former sold beef cattle shortly after they finished suckling (8 months), whereas the latter stored cattle for a second grazing season and then fattened them prior to sale. The remaining Irish case studies were dairy calf to store or beef, and store to beef or fattening operations. The latter system purchased cattle between 18 and 20 months and fattened them over a 3- to 4-month period on forage and concentrate. Similar to the suckler to beef system, the dairy calf to beef system reared and fattened cattle, but all calves were by a dairy dam. The dairy calf to store system reared cattle until the end of their second summer (16 months) and sold them to a fattening beef system.

Table 1 describes the Irish systems in more detail along with the French, Spanish and Italian farms. The French case study farms were suckler to weaning or beef with or without cattle purchases. These systems were also reliant on grazing cattle for a significant period of the year, but used less N fertilizer than the Irish farms. There was also a French case study of beef fattening systems. These systems were the most common mode of production in northern Italy and were used for four of this nation’s five case study farms. The remaining Italian farm was a suckler to beef system. The Italian beef fattening farm systems were more intensive than comparable Irish and French systems and thus produced more LW and more feeds per hectare (**ha**). In these systems, animals were reared in specialized farms where maize is the main forage source followed by hay.

**Table 1:** Technical description of French, Spanish, Italian and Irish case study beef cattle farms

Farm^1^	Grassland (Ha)	Cropland (Ha)	Cows (*n*)	Fertilizer (kg N/ha)	Concentrate (kg/head)	Cows sold (*n*)	Bulls sold (*n* (age))	Steers sold (*n* (age))	Heifers sold (*n* (age))	Net LW sold (kg/ha)
IE1	39	–	48	170	231	6	–	25 (20)	25 (24)	915
IE2	39	–	74	170	125	12	39 (8)	–	40 (8)	793
IE3	40	–	0	178	528	0	–	98 (24)	–	1353
IE4	40	–	0	178	225	0	–	140 (16)	–	1340
IE5	20	–	0	114	1001	0	–	210 (24)	–	1196
FR1	128	37	107	33	303	27	2 (>36)	52 (10)	18 (10) & 6 (36)	328
FR2	96	–	73	16	93	8	1 (>36)	35 (10)	20 (9) & 6 (36)	314
FR3	63	7	52	43	315	16	1 (>36)	64 (20)	6 (20)	733
FR4	41	–	0	100	1700	0	–	199 (18)	24	2018
FR5	60	10	68	59	174	20	–	32 (8)	9 (8) & 2 (20)	408
IT1	–	16	0	45	2100	0	–	426 (22)	–	8769
IT2	9.5	22.5	0	166	1606	0	146 (17)	–	–	1830
IT3	9	8	0	92	2303	0	–	–	206 (20)	4041
IT4	9	33	0	162	1644	0	197 (17)	–	–	1527
IT5	6	22	45	139	–	4	8 (15)	7 (9) & 3 (14)	10 (9)	363
ES1	86	157	115	–	1789	0	215 (15)	–	170 (13)	379
ES2	0	3	0	–	910	0	159 (12)	–	–	11 832
ES3	96	–	83	–	2391	0	700 (15)	–	300 (13)	1690
ES4	100	520	210	–	1861	0	490 (15)	–	210 (13)	270
ES5	0	2	0	–	3199	0	319 (12) & 424 (14)	–	–	156 875

LW = live weight.

1 Irish farms: IE1 = suckler to beef, IE2 = suckler to weaning, IE3 = dairy calf to beef, IE4 = dairy calf to store and IE5 = beef fattening. French farms: FR1-FR2 = suckler to weaning, FR3 = suckler to beef with purchases, FR4 = beef fattening and FR5 = suckler to weaning and beef. Italian farms: IT1–IT4 = beef fattening and IT5 = suckler to beef. Spanish farms: ES1, ES3 and ES4 = suckler to beef with purchases, and ES2 and ES5 = beef fattening.Suckler to weaning – beef cow progeny sold shortly after weaning (8 months); suckler to beef – beef cow progeny reared and fattened on the same farm; Beef fattening – reared cattle purchased and fattened; dairy calf to beef system – surplus calves from dairy dam(s) reared and fattened; dairy calf to store – surplus calves from dairy dam reared and sold at 10 to 20 months for fattening.

The Italian suckler to beef system is represented by a farm producing younger and lighter bulls or heifers than the similar systems in the other nations. The diet for each animal category is mainly composed of maize silage, hay and cereals produced on farm. The two Spanish case study farms that operated beef fattening systems were comparable in terms of intensity to the same farm type for Italian farms, but were very small in terms of area (i.e., both farms were <5 ha). Conversely, two of the three Spanish farms that reared sucklers to beef had the most farmland. These farms also purchased cattle for finishing. Heifers were managed extensively, and cattle for slaughter were managed intensively. The other Spanish suckler case study farm also purchased cattle for fattening and was the most intensive of the three Spanish suckler systems. The case study farms from Spain were taken from Asoprovac (2018) commercial producer group, and in Italy they were obtained from Unicarve (2018) and Asprocarne (2018) producer groups.

**Figure 1:**
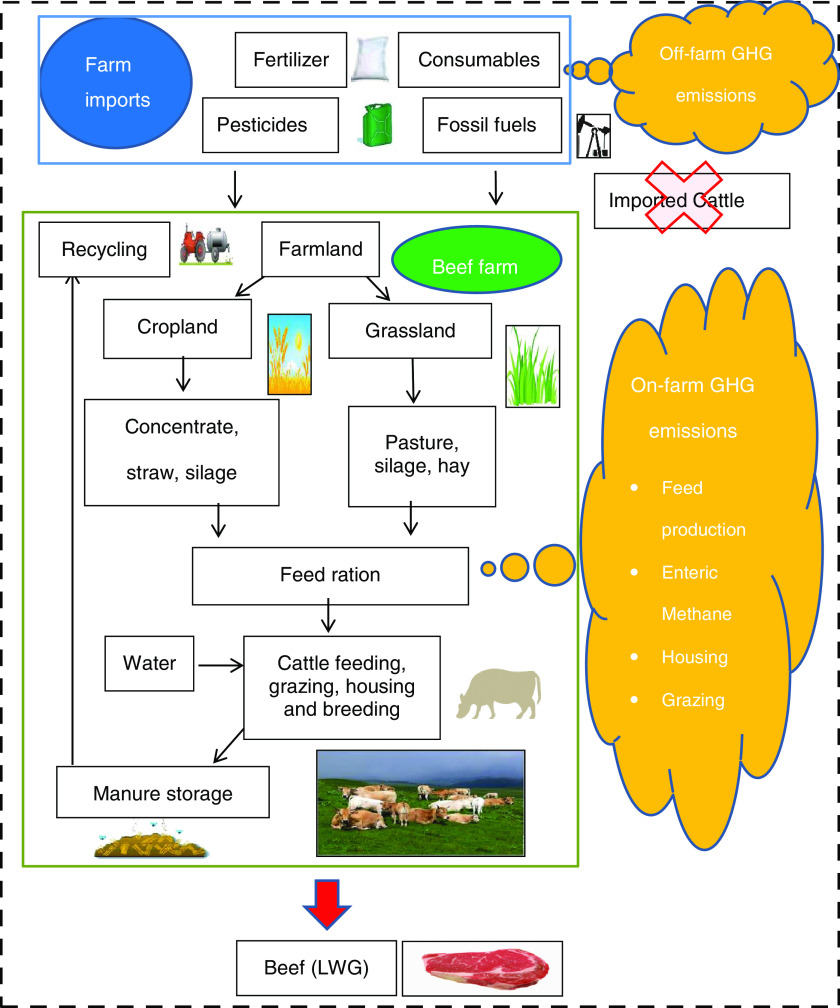
Key greenhouse gas (GHG) emission sources modelled by CAP’2ER, Carbon Audit and Bovid-CO_2_. Dotted line represents the system boundary. Blue box represents off-farm emission sources, and green box represents on-farm sources. Cattle purchases were not included.

For the case study farms, Carbon Audit, CAP’2ER and Bovid-CO_2_ focused on LWG produced on-farm and did not include GHG emissions associated with purchasing cattle, because it was not possible to estimate these emissions for all nations. Furthermore, LIFE BEEF CARBON aimed to reduce carbon emissions from cattle the producers managed. The models quantified the gross carbon footprints of beef farming systems in terms of LWG (Figure 1).

### Common mitigation plan

Measures to mitigate GHG emissions from beef farming systems vary from better animal genetics to grassland preservation to technological changes, for example, urease inhibitors. Some options are suitable on most livestock farm. For instance, increasing animal welfare and health has a positive effect on animal performance and increases resource use efficiency (Hristov *et al.*, 2013). This has positive effects on carbon footprint and other environmental categories. However, it is unlikely that all options reported to mitigate GHG emissions will be suitable to apply on commercial farms. Thus, a list of suitable measures to reduce GHG emissions and increase carbon sequestration was compiled by all partners. They were selected following a strengths, weaknesses, opportunities and threats (**SWOT**) analysis.

Strengths, weaknesses, opportunities and threats analyses were done by nations for mitigation options and entailed identifying factors that help and harm improving carbon emissions and the environment, for example, technical feasibility, farmer acceptability and economic conditions. Mitigation options that had more beneficial effects on carbon and the wider environment than negative influences were selected for the mitigation plan. The analyses utilized national studies on GHG abatement options (e.g., Lanigan *et al.*, 2018) to identify mitigation measures that could be applied on-farm. This was also determined using tools that provide decision support on mitigating beef farms’ carbon footprint, for example, Teagasc/Bord Bia Carbon Navigator (Murphy *et al.*, 2013). The outputs of the SWOT analyses were lists of GHG mitigation options suitable for each nation’s beef production systems. These lists were aggregated to develop a common mitigation plan.

## Results

### Gross carbon footprints

**Figure 2:**
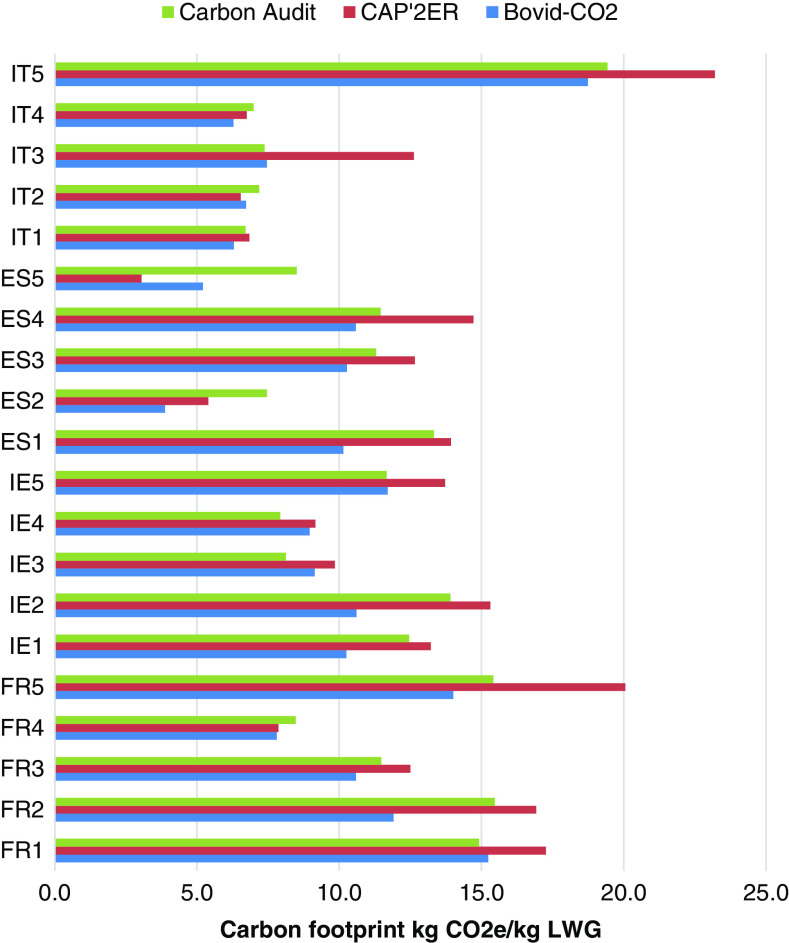
Gross carbon footprints of case study beef cattle farms calculated using the modelling tools Carbon Audit, CAP’2ER and Bovid-CO_2_. Footprints were estimated in terms of CO_2_ equivalents (CO_2_e) and related to live weight gain (LWG). French farms: FR1–FR2 = suckler to weaning; FR3 = suckler to beef with purchases; FR4 = beef fattening; and FR5 = store to weaning and beef. Irish farms: IE1 = suckler to beef; IE2 = suckler to weaning; IE3 = dairy calf to beef; IE4 = dairy calf to store; and IE5 = beef fattening. Spanish farms: ES1, ES3 and ES4 = suckler to beef with purchases and ES2 and ES5 = beef fattening. Italian farms: IT1–IT4 = beef fattening and IT5 = suckler to weaning.

Case study farm carbon footprints were initially analysed by the nations and with the same model (Figure 2). This showed suckler to weaning or store systems generally had the highest carbon footprint followed by the suckler to beef system, with the exception of the Italian suckler to beef system, because the bulls or heifers it produces are younger and lighter than the corresponding cattle of the other nations. The beef fattening system tended to have the lowest gross carbon footprint, but the difference was relatively small for the Irish case studies compared to other nations’ results. This was due to the type of animal finished, that is, bull or steer, animal age and level of supplementation. Across the case studies that analysed the complete beef farm system, that is, dairy calf to beef and suckler to beef, the former had a smaller gross carbon footprint than the latter because for dairy, most of the cows’ GHG emissions were allocated to milk instead of beef.

The ranking of nations’ beef systems’ footprints was inconsistent when a mixture of models was applied. For example, the fourth Irish case study farm, dairy calf to beef, had the lowest Irish carbon footprint using Carbon Audit. However, when CAP’2ER was used for this case study farm and Carbon Audit for the remaining Irish farms, its carbon footprint was no longer ranked the lowest.

### Model comparison

#### Carbon Audit

Carbon Audit’s average footprint estimates were slightly lower than those of CAP’2ER, but marginally higher than those of Bovid-CO2. The average difference between Carbon Audit and Bovid-CO2 gross carbon footprints was 1.2 kg of CO2e/kg LW. On average, Carbon Audit’s gross carbon footprints were lower than CAP’2ER’s by 1.1 kg of CO2e/kg LW. Across Irish and French farms, Carbon Audit showed CH_4_ from enteric fermentation was the main component of the footprint from these farms (44% to 62%). This was generally the case for Spanish and Italian farms, except the Italian case study beef fattening systems (IT3 and IT4) where concentrate contributed a greater share of GHG emissions. The remainder of a farm’s footprint, when estimated by Carbon Audit, was explained by GHG emissions from mineral fertilizer application and manufacture, manure storage and spreading, and manure deposited by grazing cattle (Table 2).

**Table 2:** Modelled carbon footprint emission profiles in percentage terms for case study beef cattle farms described in Table 1. The carbon emission source grazing returns is manure deposited by grazing cattle. The source ‘other’ includes indirect N losses, limestone and minor inputs (e.g., milk replacer)

Model	Source	IE1	IE2	IE3	IE4	IE5	FR1	FR2	FR3	FR4	FR5	ES1	ES2	ES3	ES4	ES5	IT1	IT2	IT3	IT4	IT5
Carbon Audit	Enteric methane	49.5	48.5	44.6	44.0	55.8	56.9	61.8	59.4	47.6	56.9	58.0	61.6	42.6	49.8	36.3	56.6	37.1	24.9	26.2	52.2
	Manure storage and spreading	7.6	7.1	8.0	5.3	12.1	9.2	10.1	11.1	17.3	8.8	14.9	23.8	17.1	14.1	19.0	18.2	16.4	22.1	21.8	10.3
	Grazing returns	14.6	15.8	11.1	15.5	8.5	15.2	15.0	11.1	0.0	13.7	8.7	0.0	3.8	9.9	0.0	0.0	0.0	0.0	0.0	11.3
	Concentrate	3.6	3.1	10.5	9.3	7.4	4.9	1.5	4.2	21.3	1.8	10.1	5.3	34.2	23.1	42.8	16.8	28.0	40.2	29.6	0.0
	Fertilizer	17.8	18.4	19.2	19.8	9.6	7.8	3.8	6.1	6.9	11.0	0.0	0.0	0.0	0.0	0.0	0.1	9.1	1.9	1.3	8.6
	Farm fossil Fuel use	3.0	3.0	3.2	2.8	2.8	2.5	4.3	4.9	4.6	4.4	5.8	7.6	0.3	0.5	0.5	4.7	7.4	8.5	18.8	14.2
	Other	3.8	4.1	3.4	3.2	3.8	3.5	3.5	3.2	2.1	3.4	2.5	1.7	2.0	2.6	1.4	3.6	2.0	2.3	2.3	3.4
CAP2ER	Enteric methane	61.9	61.5	59.8	61.8	63.9	63.5	64.2	67.0	56.8	66.9	47.4	41.7	35.3	49.2	8.5	57.5	46.9	37.7	38.6	59.6
	Manure storage and spreading	5.8	6.2	5.5	6.1	7.5	13.8	17.3	15.1	24.1	15.3	14.2	28.8	19.0	13.7	22.9	5.6	20.1	17.3	13.7	12.2
	Grazing returns	7.8	9.5	5.6	6.3	4.7	7.0	7.9	5.4	0.0	7.9	6.0	0.0	3.3	5.8	4.6	3.7	2.6	1.6	4.3	4.8
	Concentrate	3.1	1.5	8.5	4.4	11.5	2.6	2.9	3.5	6.3	1.3	25.0	24.0	36.0	26.5	55.0	26.6	21.7	32.8	20.0	3.5
	Fertilizer	14.3	14.3	13.4	14.5	7.0	7.6	2.8	3.1	6.5	3.5	0.0	0.0	0.0	0.0	0.0	0.4	0.0	0.8	9.4	6.2
	Farm fossil fuel use	2.8	2.9	2.6	2.8	2.4	3.2	3.7	4.3	4.8	3.2	2.9	4.0	0.3	0.4	0.4	3.3	5.3	5.3	8.7	11.4
	Other	4.2	4.0	4.6	4.1	3.1	2.3	1.3	1.6	1.6	1.9	4.4	1.6	6.0	4.4	8.5	3.0	3.5	4.6	5.3	2.3
Bovid-CO_2_	Enteric methane	59.9	61.0	60.2	63.2	63.2	65.2	64.6	53.2	48.7	50.8	43.1	40.8	37.3	44.3	26.0	54.5	52.8	33.8	29.7	56.1
	Manure storage and spreading	2.8	1.6	4.5	4.9	6.6	7.6	6.5	8.2	15.1	3.8	3.6	20.0	3.8	3.9	7.7	11.5	12.7	10.6	10.8	21.6
	Grazing returns	6.8	7.0	6.2	6.0	4.0	7.0	7.0	4.5	0.0	6.1	4.1	0.0	2.3	4.3	0.0	0.0	0.0	0.0	0.0	0.0
	Concentrate	4.7	2.8	9.7	5.0	14.6	5.3	6.3	4.7	8.1	2.6	41.9	33.0	54.8	44.6	65.1	27.4	16.8	39.5	27.3	0.0
	Fertilizer	17.5	19.0	12.3	13.7	6.7	3.6	4.7	18.5	18.4	25.3	0.0	0.0	0.0	0.0	0.0	0.3	6.8	1.5	7.0	4.9
	Farm fossil fuel use	1.9	1.7	1.5	1.5	0.7	6.8	6.7	5.1	4.8	4.6	4.6	5.2	0.4	0.5	0.6	4.8	7.7	13.2	22.5	14.6
	Other	6.5	6.9	5.5	5.7	4.2	4.4	4.2	5.8	4.9	7.0	2.7	1.0	1.3	2.3	0.6	1.5	3.2	1.3	2.6	2.8

#### CAP’2ER

In agreement with Carbon Audit, CAP’2ER showed that enteric CH_4_ was the dominant source of GHG emissions from case study farms (38% to 67%; Table 2), except for some Italian and Spanish beef fattening systems. The French model IPCC tier 3 emission factor usually estimated significantly higher enteric CH_4_ emissions per animal than Carbon Audit’s tier 2 emission factor (Supplementary Material Table S2). This emission factor difference explained most of the difference in model footprint estimates for case study farms that reared suckler or dairy calves (Figure 2). Generally, CAP’2ER showed that most of the remaining emissions were from mineral fertilizer and manure (Table 2).

For some Italian and Spanish beef fattening systems CAP2ER indicated that concentrate was a key source of GHG emissions or the main source. Carbon Audit estimated lower or similar carbon footprints than CAP’2ER for Italian beef fattening systems. For similar Spanish beef systems CAP’2ER tended to estimate lower footprints than Carbon Audit.

#### Bovid-CO_2_

Bovid-CO_2_ estimates of farm enteric CH_4_ emissions were generally lower than those of Carbon Audit and CAP’2ER and thus resulted in lower gross carbon footprint estimates for this model (Figure 2). The Spanish tool showed that the remaining beef farm GHG emissions were normally from mineral fertilizer, manure excreted by grazing cattle and emissions from manure storage and spreading for Irish and French farms (Table 2). Concentrate production was generally a minor component of case study farms’ carbon footprint, but Bovid-CO_2_ tended to apply a higher emission factor for this source than the other model and thus estimated a higher concentrate emission. This effect was magnified for Spanish or Italian farms where concentrate was fed *ad libitum* in fattening beef system, for example, concentrate accounted for 65% of ES5 case studies’ farm gross carbon footprint. This was the highest relative estimate for this source between the three models.

### Mitigation strategies

The typical outputs of the SWOT analysis are shown in Table 3 for the mitigation option maintaining and/or planting hedgerows or trees. This option’s SWOT showed potential positive impacts outnumbered negative impacts and was thus selected for the mitigation plan. The SWOT was repeated for all mitigation options and used to create a common or beef carbon mitigation plan (Table 4). The plan shows the effect these options have on CH_4_, N_2_O, CO_2_ and their potential to mitigate net carbon footprint of beef, which varied from <1% to 15%. Generally, animal performance and diet options had the greatest GHG mitigation potential, followed by the soil and land-use options. The manure mitigation options’ potentials were intermediate, and the energy mitigation options had the least potential. The options nations prioritized from the common mitigation plan are shown in Supplementary Material Table S3 and reflect the type of beef farm system(s) in operation and local production circumstances, for example, weather conditions and market prices.

**Table 3:** Beef carbon mitigation options’ potential strength, weaknesses, opportunities and threats (SWOT)

Measure : Maintain or plant hedgerows and/or trees	
Help carbon emission and environment	Harm carbon emission and environment
*Strengths* – Easy to implement – Protect or increase soil carbon – Enhance farm biodiversity – Provide shelter – Displace fossil fuel – Reduce beef carbon footprint by 3% to 10%	*Weaknesses* – Habitat for carriers of animal diseases, for example, ticks – Increase production costs – Farm fire hazard
*Opportunities* – Diversify landscape – Improve sustainability – High value food market – Wood products – Create alternative employment – Develop rural economy	*Threats* – Extreme weather events – Uncertainty regarding rate and permanence of carbon storage – Mitigation allocated to different sector(s), for example, energy and transport

**Table 4:** Effect of mitigation strategies on beef farming systems’ greenhouse gas (GHG) emissions and net carbon footprint (i.e., GHG emission/unit of live weight gain (LWG))^1^

Mitigation strategies	Greenhouse gases^2^			Net carbon footprint^3^
	CO_2_	CH_4_	N_2_O	
Animal performance				
Increase average daily weight gain	±	−	−	−3% to −10%
Reduce slaughtering age	±	–	–	−5% to −10%
Improve animal health	–	–	–	−5% to −15%
Optimize age at first calving (e.g., 24 months)	–	–	–	−5% to −10%
Optimize calving rate (e.g., 0.95 to 1 calf/cow)	–	–	–	−5% to −10%
Improve genetic merit	–	–	–	−2% to −10%
Diet				
Improve grassland management (e.g., adopt rotational grazing)	–	–	+	−3% to −10%
Increase forage quality	±	−	−	−3% to −8%
Increase fraction of concentrate in the diet	+	–	±	−15% to +20%?
Optimize concentrate crude protein content	–	±	–	−3% to −8%
Replace soy meal with low emission alternatives (e.g., rapeseed meal)	–	±	±	−3% to −15%?
Feed agro-alimentary by-products	–	±	–	0% to −5%?
Feed additives (e.g., lipids, yeast, nitrate amino acids)	±	–	±	−15% to +5%?
Soil fertility and N fertilizer				
Improve soil pH via liming	+	–	–	−2% to −5%
Optimize soil N, P and K levels	+	–	–	0% to −5%
Optimize mineral N fertilizer application via precision technologies (e.g., GPS)	–	±	−	−2% to −5%?
Incorporate legumes into the sward (clover)	–	–	–	−2% to −10%?
Replace mineral fertilizer with manure	–	+	±	−2% to −5%?
Change from CAN to Urea fertilizer	±	±	–	−2% to −5%
Management of stored manure				
Extend length of grazing season	–	±	+	0% to −8%
Cover manure store (e.g., UV-stabilised plastic covers, peat, straw or wood chips)	±	−	±	−2% to −5%?
Store solid manure on solid impermeable floor equipped with a drainage system	±	±	−	−0% to −3%
Anaerobic digestion/biogas	±	–	–	−3% to −10%
Aeration	±	–	±	0% to −5%?
Composting	±	–	±	−2% to −5%
Partial/total replacement of deep litter with fully slatted floor	±	–	+	0% to −1%
Install fans to reduce straw bedding	–	±	±	0% to −1%
Air cleaning systems (e.g., scrubbers)	+	±	–	0% to −5%
Manure treatment				
Nitrification inhibitor	±	±	–	0% to −5%?
Urease inhibitor	±	±	−	0% to −5%?
Acidification	±	–	±	0% to −5%?
Solid separation	±	–	±	0% to −5%?
Low emission slurry spreader	±	±	–	−2% to -5%
Manure injection (Rapid incorporation)	–	±	+	0% to −1%
Energy				
Increase renewable energy use (e.g., solar)	–	±	±	−1% to −2%?
Low energy lighting	–	±	±	0% to −1%
Meter equipment’s electricity consumption	–	±	±	−1% to −2%
Match tractor power to field work task	–	±	±	−1% to −2%?
Carbon sequestration				
Preserve or increase permanent grassland area	–	±	±	−3% to −10%?
Maintain or plant hedgerows/trees	–	±	±	−3 to −10%?
Minimum or no till	–	±	–	0% to −5%

GPS = global positioning system; CAN = calcium ammonium nitrate.

1 Live weight gained by beef cattle produced on-farm.

2 Change in CO_2_, methane (CH_4_) and nitrous oxide (N_2_O) emissions per unit of LWG. Negative sign indicates a reduction in emission intensity; positive sign indicates an increase and ± indicates a mixed or no effect.

3 Change in net carbon footprint estimated from national research studies and from United Nations Food and Agriculture Organization (FAO) reports (Hristov *et al.*, 2013; Montes *et al.*, 2013). Measures with ? indicate high uncertainty in the level of reduction.

The high mitigation potential of the animal performance strategy was due to increases in production efficiency. This strategy dilutes GHG emissions, that is, maintains LWG and reduces emissions, particularly enteric CH_4_. For example, improving animal health reduces cattle mortality rates and thus the requirement for extra cows, which results in lower GHG emissions for the same level of product. Dietary options can also have this productivity effect, and some (e.g., feed additives) can potentially directly reduce the enteric CH_4_ loss factor to <5% of gross energy intake. This option was among the measures that had the greatest potential to mitigate beef carbon footprint. However, the level of uncertainty in the reduction was high compared to animal performance options. This was also an issue for promising soil and land-use options that displaced N fertilizer (e.g., white clover) or increased soil carbon (e.g., preserving grassland).

## Discussion

Beef farms’ carbon footprints are inherently uncertain. This is caused by animal, soil and plant variability and by inconsistent modelling methods and decisions. The latter further complicates different beef LCA modelling studies, but when sufficient information is available, model comparisons can be used to determine the likely emissions from beef systems and the efficacy of mitigation measures. Moreover, model comparisons are useful to validate outcomes, where measurement is challenging. This is especially pertinent for livestock CH_4_ emissions and soil N_2_O emissions.

The models selected for LIFE BEEF CARBON were validated against widely published models such as GLEAM (Opio *et al.*, 2013) using the outcomes from case study farms. This showed the results of Carbon Audit, for the respective countries, were in the mid to lower range of reported carbon footprints, except for French suckler to weaning farms that were in the higher end of the national range (Supplementary Material Table S1). Carbon footprints quantified by CAP’2ER for French and Irish farms were generally in the mid or upper half of the reported national ranges. CAP’2ER footprints were in the lower to middle range of values for comparable Italian studies. The models Spanish farm results were in the middle or lower range of reported national values, except one farm that was below the lower bound.

The Spanish models’ (Bovid-CO_2_) carbon footprints for case study farms were below or in the lower range of reported national values for beef fattening systems or suckler to beef farms. Bovid-CO_2_ footprint results for Italian and Irish farms were normally in the lower and middle range for both nations. The tools’ results for French farms ranged from low to high when compared to French studies. Across suckler to beef case study farms, Bovid-CO_2_ beef carbon footprints were below the global average reported by Opio *et al.* (2013) for the full life cycle of beef cattle (Table 5). Excluding the CAP’2ER footprint for the Italian suckler to beef system, the other models’ case study suckler to beef footprints were below the global average as well, mainly because all farms were located in developed nations and generally more efficient than a country’s typical producer. Nevertheless, there was still a considerable range between similar case study farms’ beef carbon footprints, which suggest there is scope to mitigate their footprints via improving productivity.

**Table 5:** Case study^1^ and UN FAO GLEAM^2^ beef carbon footprints for suckler to beef and dairy calf to beef farm systems

Beef system	LCA^3^ model	Carbon footprint
FR3 – suckler to beef	CAP’2ER	12.5
IE1 – suckler to beef	Carbon Audit	12.5
IE3 – dairy calf to beef	Carbon Audit	8.1
ES1 – suckler to beef	Bovid-CO2	10.2
ES3 – suckler to beef	Bovid-CO2	10.3
ES4 – suckler to beef	Bovid-CO2	10.6
IT5 – suckler to beef	CAP’2ER	23.2
Suckler to beef average	As above	13.2
Global – suckler to beef	GLEAM	37.3
Western Europe – suckler to beef	GLEAM	32.0
Global – dairy calf to beef	GLEAM	10.1
Western Europe – dairy calf to beef	GLEAM	7.7

1 Described in Table 1.

2 United Nations Food and Agriculture Organization global livestock environmental assessment model (Opio *et al.*, 2013).

3 Life cycle assessment.

The modelling approach we applied, LCA, can capture most, if not all, emission reduction strategies for beef production, but relative to the national GHG inventory (IPCC) approach, LCA attributes more emissions to beef, for example, imported feed emissions (Crosson *et al.*, 2011). In addition, these sources add more uncertainty to beef farms’ GHG emission estimates and require extra resources to gather and collate data across nations. This can be particularly challenging in developing countries. The inventory or IPCC method focuses on national emissions only. Thus, tracing the origin of imported goods is not an issue, but this national approach can lead to carbon leakage, for example, the transfer of beef production to regions with a larger beef carbon footprint. It is also more difficult to improve national inventories and takes longer compared to LCA. Consequently, the national inventory method does not capture the true mitigation potential of the beef sector.

### Common modelling framework

Applying Irish, French and Spanish modelling tools to compare the GHG emissions of case study farms showed that the LCA models estimated different gross carbon footprints for the same farms. This finding implies it is not valid to use a mixture of models to compute carbon footprints within a sample or group of farms. Ideally the same GHG or footprint model should be used when analysing footprints between farm groups, but this may not be appropriate when a model is not adapted to local production systems. Thus, other GHG models may be needed.

The three models compared accounted for the same GHG sources using IPCC or national guidelines and used a common modelling approach, that is, cradle to farm-gate LCA and whole farm models. Therefore, the differences between models were due to the emission factors and equations used to estimate farm GHG sources. Berton *et al.* (2016) demonstrated that these calculations can have a major impact on beef farms emissions, particularly enteric CH_4_. This source is the main determinant of beef farms’ gross carbon footprint (Dollé *et al.*, 2011; Opio *et al.*, 2013) and is dependent on the type and quality of feed cattle offered. Enteric CH_4_ is best estimated using locally adapted emission factors. The models follow this approach when applied to national or regional production systems, but not for other regions systems. This problem could be overcome by adapting models to other countries’ farms, but they are rarely applied outside their region or country of origin, for example, Carbon Audit is not used in Italy. Thus, the original geographical coverage of their enteric CH_4_ emission factors was not expanded.

Concentrate production is a key source of GHG emissions for beef fattening systems (Doreau *et al.*, 2011; Boselli *et al.*, 2015; Berton *et al.*, 2016). The emission factors for this source is usually difficult to estimate as the quantity of ingredients in concentrate feedstuffs, particularly compounds, is often unknown. Furthermore, the emissions from these ingredients vary between regions and nations due to methods of cultivation and processing, and natural factors, that is, weather, soil type, topography and altitude. It is, however, possible to account for some of this variation by adding more concentrate emission factors to models and widening their geographical coverage. This was considered for some models that are applied outside their national context. The remaining key GHG emissions sources were calculated by CAP’2ER, Carbon Audit and Bovid-CO_2_ using similar emission factors and thus did not require updating. CAP’2ER was the only model that originally considered carbon sinks like grassland.

The approach CAP’2ER uses to estimate carbon sequestration by soil was assessed by Carbon Audit and Bovid-CO_2_ practitioners and subsequently added to these models using the results of sequestration research carried out on national field sites. This update will facilitate the calculation of a net carbon footprint by the models for the commercial farms part of LIFE BEEF CARBON. The updated modelling tools developed will be applied to assess gross and net carbon footprints of farms within their geographic coverage, which was usually the national level. Furthermore, the models were expanded, where necessary, to assess other key environmental metrics, for example, biodiversity in order to avoid implementing carbon reduction actions that have negative environmental effects. The models use the same approaches that are reported by CAP’2ER to quantify these other key environmental metrics, namely, acidification, eutrophication, non-renewable energy use and land occupation. Consequently, we did not compare these indicators for the case study farms.

Overall, this framework facilitates a common approach to assess the environmental performance of beef farms and accounts for the different production contexts in each nation or region. This wider framework provides a relatively simple and complete environmental assessment and can quantify the directional change in farm’s GHG emissions.

### Reducing beef carbon

LIFE BEEF CARBON overarching goal is to reduce commercial farms’ beef carbon footprint by 15% over 10 years. This study analysed the potential to achieve this goal by quantifying the mitigation potential of an array of options reported in the national and international literature. Consistent with Lanigan *et al.* (2018), Bellarby *et al.* (2013) and Pellerin *et al.* (2017), our analysis showed many options are available to reduce beef carbon footprint, but no single option is sufficient by itself (i.e., single mitigation options reduced the beef footprint by less than the overall target). However, some of the mitigation options identified can be applied together and are additive or synergistic. The combined mitigation potential of these additive options indicated the LIFE BEEF CARBON footprint reduction goal can be achieved or surpassed for all partner nations.

Examples of reliable options that can be simultaneously implemented to substantially reduce beef carbon footprint include improving animal productivity, increasing forage quality and enhancing soil fertility and fertilizer use efficiency. These synergistic mitigation options positively influence farm finances and are therefore likely to be adopted by farmers. They also tend to have positive affect on other important environmental impact measures, for example, ammonia (Asem-Hiablie *et al.*, 2018). All partners aim to improve these efficiency and productivity mitigation options on commercial beef farms along with maintaining or increasing soil carbon. The commonly recommended options to build soil carbon, for example, planting hedgerow and preserving grassland are expected to complement on-farm efficiency measures. In addition, preserving these habitats conserves cultural landscapes and enhances biodiversity (Dollé *et al.*, 2013; Bragaglio *et al.*, 2018).

Further mitigation of emissions is possible on-farm by using new plant species, particularly white clover, and low emission technologies, for example, urease inhibitors (Lanigan *et al.*, 2018). These mitigation options are likely to be positive for biodiversity, but may not be additive for GHG emissions, as they have similar effects on the same emission source, mineral N fertilizer. Some low emission technology options may also cause increases in N_2_O emission that outweigh the benefits of reductions in other GHG emissions and reduce profitability, for example, aeration during slurry storage (Montes *et al.*, 2013). A holistic approach is thus needed when evaluating new farm machinery (inputs) to prevent emission transfers and to avoid safety and product residue issues that could reduce the sustainability of beef farming.

## Conclusions

The selected modelling tools provide a common methodological framework to quantify beef carbon footprints, when applied in the nation(s) they are adapted to. These models capture the effect of farm system on beef’s carbon footprint and the influence of local production circumstances, for example, climate and soil type(s). In addition, they can evaluate carbon footprint mitigation strategies and, following updates carried out to Carbon Audit and Bovid-CO_2_, estimate other environmental impacts. This wider assessment should reduce the risk of implementing GHG emission mitigation strategies that simply transfer pollutants from one environmental impact to another. It also facilitates better comparisons between partner nations’ beef production systems and more opportunities to exchange carbon footprint mitigation options. The estimated mitigation effect of all nations’ carbon footprint reduction plans meets the LIFE BEEF CARBON target. These plans will be tested on 172 innovative farms across the four nations to determine what works on commercial farms. These results will be used to create action plans to reduce beef carbon footprint on farms in Western Europe.

## Software and data repository resources

Microsoft Excel was used to store data and model farms. Access to these models was limited to developers.

## Supplementary material

For supplementary material accompanying this paper visit https://doi.org/10.1017/S1751731119002519. click here to view supplementary material
