# Supplementary material for: LIFE BEEF CARBON: a common framework for quantifying grass and corn based beef farms’ carbon footprints
Source: Animal. 2019 Oct 31;14(4):834–45. doi: 10.1017/S1751731119002519 (PMC7283046; doi:10.1017/S1751731119002519)
Supplement: Supplementary file 1 [file S1751731119002519sup001.docx]

***animal*** journal

LIFE BEEF CARBON: A common framework for quantifying Western European beef farms carbon footprints

D. O’Brien, J. Herron, J. Andurand, S. Caré, P. Martinez, L. Migliorati, M. Moro, G. Pirlo, and J-B. Dollé

**Supplementary material**

Supplementary Table S1 Inventory analysis of studies carried out in Ireland, France, Spain and Italy modelling greenhouse gas (GHG) emissions from beef farming systems

| Nations | Project type | Model type and study goal(s) | Farm description and carbon (C) footprint^1^ | GHG Emission factors | Other impacts |
| --- | --- | --- | --- | --- | --- |
| Ireland | National research project | Casey and Holden (2006) developed a cradle to gate life cycle assessment (LCA) model for suckler beef systems – The studies main aim was to estimate GHG emissions from a typical beef farm. The study also tested mitigation strategies using scenario analysis. | National average farm gross C footprint  11.3 kg CO_2_e/kg LW. | IPCC (1996) and literatures sources | No |
| Ireland | National research project | Foley et al. (2011) developed BEEFGEM a whole farm GHG model for suckler beef – The goals of this study were to assess the GHG emissions and profitability of alternative suckler beef systems. | National average farm gross C footprint  23.1 kg CO_2_e/kg CW.  Research farms gross C footprints scenario range  18.9-22.0 kg CO_2_e/kg CW. | Ireland national GHG inventory method, IPCC (2006) and literatures sources | No |
| Ireland | National research project | Clarke et al. (2013) developed a cradle to farm-gate LCA model for suckler beef – The study examined GHG emissions from grass-based beef farms differing in stocking rate and type of male i.e. bull or steer. | Research farm gross C footprints scenario range  20.1-23.1 kg CO_2_e/kg CW. | Ireland national GHG inventory method, IPCC (2006) and literatures sources | No |
| Ireland | National research project | Crosson et al. (2013) developed Carbon Audit, which is a certified whole farm GHG model constructed from BEEFGEM – This studies goal was to quantify GHG emissions from commercial suckler and dairy calf to beef farms with the Irish beef industry. | 200 commercial farms part of the beef quality assurance scheme – 32 000 farms are part of this scheme and are audited every 18 months.  ^2^Gross C footprints range 9.0-14.6 kg CO_2_e/kg LW. | Ireland national GHG inventory method, IPCC (2006) and literatures sources | No |
| Ireland | National research project | Murphy et al. (2017) applied BEEFGEM a whole farm GHG model for dairy calf to beef systems – The goal of this research was to quantify the effect of diet and slaughter age on GHG emissions and farm profitability. | Research farm gross C footprints range  8.9-14.9 kg CO_2_e/kg CW. | Ireland national GHG inventory method and literature sources | No |
| France | National research project | Dollé et al. (2011) used GES’TIM a cradle to farm-gate LCA model for French livestock production that is part of the certified tool CAP’2ER – The aim of this review study was to estimate carbon footprints for French beef and sheep meat. | 5 farms representative of national beef farm systems.  Gross C footprints range 11.3-14.0 kg CO_2_e/kg LW.  ^3^Net C footprints range  6.5-8.5 kg CO_2_e/kg LW. | French national GHG inventory, IPCC (2006) and literature sources | Yes – Non-renewable energy use |
| France | National research project | Doreau et al. (2011) developed a gate to gate LCA model for beef finishing systems – Goal of this research was to estimate the effect different diets have on GHG emissions from bull beef finishing systems. | Research farm. Gross C footprints range  3.7-5.2 kg CO_2_e/kg LWG.  Net C footprints range  3.7-4.6 kg CO_2_e/kg LWG. | Methane emission from cattle was measured and manure emissions were based on IPCC (2006). Literature was used for other sources | No |
| France | National research project | Veysset et al. (2014) used GES’TIM a cradle to farm-gate LCA model for livestock production that is now part of CAP’2ER – Aim was to assess GHG emissions and non-renewable energy use from real-world French farm businesses. | 59 commercial farms. Gross C footprints range 11.0-14.7 kg CO_2_e/kg LW.  Net footprint ranged from  8.0-12.1 kg CO_2_e/kg LW. | French national GHG inventory, IPCC (2006) and literature sources | Yes – Non-renewable energy use |
| Italy | European research project; LIFE+ Climate changE-R | Emilia Romagna Agriculture and Fishing (2018) developed a cradle to farm-gate LCA model for beef systems – The aim of this research was to quantify the environmental performance of beef cattle from the Emilia-Romagna region. | 8 commercial Italian farms in Emilia-Romagna. Gross C footprints range  8.8-13.5 kg CO_2_e/kg LW. | Italian national GHG inventory, IPCC (2006) and literature sources | No |
| Italy | National research project | Boselli (2015) applied CAP’2ER cradle to farm gate LCA model for Italian beef systems – The main goal of this research was to estimate the environmental impact of breeding and finishing beef production systems in Northern Italy. | 30 commercial farms in northern Italy. Gross C footprints range  15.2-37.7 kg CO_2_e/kg LW finished. | French national GHG inventory, IPCC (2006) and literature sources | Yes – Acidification, Eutrophication, Energy demand, biodiversity, water footprint |
| Spain | National research project | Batalla et al. (2014) developed a cradle to farm gate LCA model for Spanish livestock systems – The goal of this study was to estimate a group of Spanish beef farms GHG emissions. | 5 commercial beef farms in Andalusia. Gross C footprints range  15.3-48.1 kg CO_2_e/kg CW. | IPCC (2006) and literature sources | No |
| Spain | European research project; Regen farming | del Hierro et al. (2017) developed NAIA a cradle to farm gate LCA model for Spanish livestock systems – The goal of this research was to describe a model to estimate the GHG emissions from meat produced by ruminant livestock in the Basque country. | Model farm in the Basque region. Beef C footprint was not estimated. | Spanish national GHG inventory, IPCC (2006) and literature sources | No |
| All | European research project; Animal Change | Hutchings et al. (2013) developed a farm model to simulate GHG emissions from livestock systems – The aim of this research was to develop a model to simulate GHG emissions from ruminant production systems in Europe, Africa and South America. | National or regional representative livestock farms. Beef C footprint was not estimated. | IPCC (2006) and literature sources | No |

^1^ Carbon footprint was estimated in kg of CO_2_ equivalent (CO_2_e) and related to live weight (LW), live weight gain (LWG) or carcass weight (CW).

^2^ Gross carbon footprint excludes the removal of carbon by soil (carbon sequestration).

^3^ Net carbon footprint includes the removal of carbon by soil (carbon sequestration).

Supplementary Table S2 Key nitrogen and greenhouse gas emission factor computations of selected beef farm modelling tools – Carbon Audit, CAP’2ER and Bovid-CO_2_.

| Emission factor (EF) | Carbon Audit | CAP’2ER | Bovid-CO_2_ |
| --- | --- | --- | --- |
| N excretion | N excretion (kg/d) = N excreted dung + N excreted urine N excreted dung = (N intake × 0.262) + 0.0091 (Yan et al. 2007). N excreted urine = (N intake × 0.467) - 0.0008 (Yan et al. 2007).  N intake (kg/d) = Feed intake × N concentration of the diet | Default values per animal category adjusted based on the time housing or grazing (Nitrates Directive; European Council, 1991) | Default value per animal category. This was the same for each farm (MAPAMA, 2017) |
| N leaching | N leaching (kg/yr) = (N from fertilizer spreading + N from manure excreted by cattle on pasture + N available from stored manure) × 0.1  (Duffy et al., 2014) | N leaching = N inputs - N outputs + symbiotic fixation + atmospheric deposition - N storage - N volatilization  N inputs = Mineral fertilizers + Concentrate feeds N outputs = live weight | Not taken into account |
| Methane (CH_4_) from enteric fermentation | CH_4_ estimated in MJ/d for categories  Grazing = 6.5% of gross energy intake (GEI^1^; Tier 2^2^; IPCC, 2006)  Indoors = DEI^3^ × [0.096 + 0.035 SDMI/TDMI^4^] - 2.298 (FL^5^ - 1)  (Tier 2; Yan et al., 2000)  Concentrate >90% of DM^6^ diet = 3% of GEI (Tier 2; IPCC, 2006) | g CH_4_/kg digestible organic matter = 45.2 – 6.66 × NI + 0.75 × NI2 + 19.65 × PCO – 35 × PCO - 2.69 × NI × PCO where NI = feeding level expressed as a % of BW. PCO = proportion of concentrate feeds in the DM diet.  (Tier 3; Sauvant et al., 2016) | EF dependant on GEI and digestibility. EF adapted from IPCC (Tier 2; MAPAMA, 2017) |
| CH_4_ from solid manure | kg CH_4_/animal category per yr = Manure VS excreted^7^ × 0.67 × B_0_^7^ × ((MCF1 × MS1) + (MCF2 × MS2)) where B_0_ = 0.24, MCF^8^ for solid manure system (FYM) is 0.02 and 0.17 for slurry, and MS is proportion of manure handled in storage system (Tier 2 IPCC, 2006; Met Eireann 2013) | Emissions factors for animal categories calculated with IPCC (2006) Tier 2 equation (Non-digestible organic matter × 0.67 × B_0_ × MCF). | Emissions factors by animal category depending on the temperature (Tier 1; IPCC, 2006) |
| CH_4_ from manure spreading | CH_4_ emission from manure spreading negligible (Chadwick et al., 2000). | Not taken into account | Not taken into account |
| CH_4_ from manure deposited by grazing cattle | kg CH_4_/animal category per yr = Manure VS excreted × 0.67 × B_0_ × MCF  where MCF = 0.01 and B_0_ = 0.24  (Tier 2; IPCC, 2006; Met Eireann, 2013) | Emissions factors for animal categories calculated with IPCC (2006) Tier 2 equation (Non-digestible organic matter * 0.67 × B_0_ × MCF). | Not taken into account |
| Nitrous oxide (N_2_O) from stored manure  * EF dependant on the manure storage system and nitrogen excretion | Solid storage: 0.02 kg of N_2_O -N/kg of N excreted. Slurry with natural crust cover: 0.005 kg of N_2_O-N/kg of N excreted (Tier 2; IPCC, 2006). | N_2_O-N emission calculated as function of manure N stored.  Solid storage: 0.5% of N excreted. Slurry with natural crust cover: 0.5% of N excreted. Pit storage below animal confinements: 0.2% of N excreted Deep bedding with no mixing: 1% of N (Tier 2; IPCC, 2006) | N_2_O-N emission calculated as function of manure N stored.  Solid storage: 0.5% of N excreted.  Slurry with natural crust cover: 0.5% of N excreted.  Pit storage below animal confinements: 0.2% of N excreted. Deep bedding with no mixing: 1% of N excreted  (Tier 2; IPCC, 2006) |
| N_2_O from manure spreading | 1% of manure N spread in solid and slurry form emitted as N_2_O–N  (Tier 1; IPCC, 2006). | 1% of N spread whatever the manure type (solid manure or slurry) emitted as N_2_O. (Tier 1; IPCC, 2006) | 1% of N spread whatever the manure type (solid manure or slurry) emitted as N_2_O.  (Tier 1; IPCC, 2006) |
| N_2_O from mineral fertilizer spreading | Regardless of the fertilizer type 1% of mineral N spread emitted as N_2_O-N  (Tier 1; IPCC, 2006). | 1% of N mineral spread whatever the fertilizer type emitted as N_2_O-N  (Tier 1; IPCC, 2006) | 1% of N mineral spread whatever the fertilizer type emitted as N_2_O -N (Tier 1; IPCC, 2006) |
| N_2_O from manure deposited by grazing cattle | EF urine = 0.02 kg N_2_O-N /kg N EF faeces = 0.02 kg N_2_O-N /kg N (EF urine × N urine + EF faeces × N faeces) × 1.5 (Tier 1; IPCC, 2006). | EF urine = 0.015 kg N_2_O-N /kg N EF faeces = 0.004 kg N_2_O-N /kg N  (EF urine × N urine + EF faeces × N faeces) × 1.5 (Oenema et al., 1997). | 1% of the total N excreted by animals grazing pasture emitted as N_2_O -N. (Tier 1; IPCC, 2006) |
| N_2_O crop residues | 1% of N input from crop residues (Tier 1; IPCC, 2006). Not included for permanent grassland. | Not considered | 1% of N input from crop residues (Urbano, 2010; Dominguez, 1997; IPCC, 2006) |
| N_2_O temporary grassland ploughing | Not considered for temporary grassland. Assumed stable | N released calculated according to sequestration/releasing of C: C/N = 10 1% of N released (Tier 1; IPCC, 2006). | Not considered |
| N_2_O from N that is re-deposited after leaching | 0.75% of N leached from N inputs emitted as N_2_O-N (Tier 1; IPCC, 2006). | 0.75% of N leached from N inputs emitted as N_2_O -N. (Tier 1; IPCC, 2006) | 0.75% of N leached from N inputs emitted as N_2_O -N.  (Tier 1; IPCC, 2006) |
| N_2_O from N that is re-deposited after volatilization | 1% of ammonia (NH_3_) volatilized from N inputs (Tier 1; IPCC, 2006).  Ammonia = Housing + grazing + manure spreading + mineral fertilizer application  Housing - Slats 0.01-0.04 kg NH_3_/animal per day. Bedded 0.01-0.03 kg NH_3_/animal per day (Hyde et al., 2003).  Grazing - 20% of N excreted on pasture emitted as NH_3_ (Tier 1; IPCC, 2006)  Manure spreading - 26-48% of TAN^9^ for slurry and 81% of TAN for solid manure (Hyde et al., 2003, Duffy et al., 2015)  Fertilizer - 2% of N for ammonium-based fertilizer and 23% of Urea fertilizer N (Misselbrook et al., 2007) | 1% of ammonia (NH_3_) volatilized from N inputs. (Tier 1; IPCC, 2006)  N volatilized is calculated with NH_3_ and nitrogen oxide (NO) emissions from mineral N and manure spreading: 10% of NH_3_ and NO emitted from N mineral spreading are re-deposited 20% of NH_3_ and NO emitted from manure spreading are re-deposited | 1% of ammonia (NH_3_) volatilized from N inputs. (Tier 1; IPCC, 2006)  N volatilized is calculated with NH_3_ and NO emissions from N minerals and manure spreading: 10% of NH_3_ and NO emitted from N mineral spreading are re-deposited 20% of NH_3_ and NO emitted from manure spreading are re-deposited |
| CO_2_ equivalent (CO_2_e) fuel combustion | 3.02 kg CO_2_e/litre of diesel  (Carbon Trust, 2013). | 3.25 kg CO_2_e/ litre of fuel | 2.98 kg CO_2_e/litre of diesel  2.97 kg CO_2_e/litre of gasoline  (CNMC, 2017) |
| CO_2_ limestone application | 0.12 kg CO_2_-C/kg limestone  (Tier 1; IPCC, 2006). |  | 0.12 kg CO_2_-C/kg limestone |
| CO_2_e from electricity | 0.60 kg CO_2_e/kWh^10^  (Howley et al., 2011). | 0.055 kg CO_2_e/ kW | 0.15-0.36 kg CO_2_e/kWh  (MAPAMA, 2017) |
| CO_2_e from making mineral fertilizer and lime | Mineral N: 7.11 kg CO_2_e/kg N  P fertilizer: 1.85 kg CO_2_e/kg P  K fertilizer: 1.77 kg CO_2_e/kg K  Lime: 0.15 kg CO_2_e/kg  (Carbon Trust, 2013). | Mineral N is only considered: 5.36 kg CO_2_e/kg N | Mineral N^11^: 3.15-6.17 kg CO_2_e/kg N (Gac et al., 2011)  Lime: 0.0116 kg CO_2_e/kg |
| CO_2_e from producing compound concentrate feedstuffs | 16% crude protein (CP) without soybean meal: 0.18 kg CO_2_e/kg DM.  17% CP with soybean meal:  0.87 kg CO_2_e/kg DM.  12.5% CP without soybean meal:  0.24 kg CO_2_e/kg DM. (Ecoinvent 2010; Carbon Trust, 2013). | 0.76 kg CO_2_e/kg DM and 1.579 kg CO_2_e/kg DM (soy cake). Purchased forages not considered | 0.5-0.6 kg CO_2_e/kg DM (dependant on concentrate CP).  1.58 kg CO_2_e/kg DM soy cake. |
| Carbon sequestration | Not considered. Model was updated to include permanent grassland as a sink: 370 kg C/ha (Soussana et al., 2010). | Permanent grassland: 570 kg C/ha.  Hays: 125 kg C/100 ml.  Mountain pasture: 250 kg C/ha.  Temporary grassland: 80 kg C/ha Other crops not in rotation: 160 kg C/ha | Not considered. The emission factors provided by CAP2ER were adopted. |

^1^ GEI = Gross energy intake ^2^ Tier 2 or higher emission factors are country specific. Tier 1 or default emission factors are IPCC (2006) global or continental averages ^3^ DEI = Digestible energy intake ^4^ SDMI/TDMI = Silage dry matter intake/total dry matter intake ^5^ FL = Feeding level above maintenance energy requirement ^6^ DM = Dry matter ^7^ B_o_ = Manure volatile solids maximum methane potential ^8^ MCF = Methane conversion factor ^9^ TAN = Total ammoniacal nitrogen ^10^ kWh = kilowatt hour ^11^ Emission factor dependant on type of fertilizer e.g., urea or ammonium fertilizer

Supplementary Table S3 Options selected by nations to mitigate beef farming systems net carbon footprint i.e. greenhouse gas emission/unit of live weight gain (LWG).

| Mitigation strategies | Ireland | France | Spain | Italy |
| --- | --- | --- | --- | --- |
| Animal performance |  |  |  |  |
| Increase average daily weight grain | ✓ | ✓ | ✓ | ✓ |
| Reduce slaughtering age | ✓ | ✓ | ✓ | ✓ |
| Improve animal health | ✓ | ✓ | ✓ | ✓ |
| Optimize age at 1^st^ calving e.g., 24 months | ✓ | ✓ | ✓ | ✓ |
| Optimize calving rate e.g., 0.95 to 1 calf/cow per year | ✓ | ✓ | ✓ | ✓ |
| Improve genetic merit | ✓ | ✓ | ✓ | ✓ |
|  |  |  |  |  |
| Diet |  |  |  |  |
| Improve grassland management e.g., rotational grazing | ✓ | ✓ | ✓ | × |
| Increase silage or hay quality | ✓ | ✓ | ✓ | ✓ |
| Increase fraction of concentrate in the diet | × | × | ✓ | ✓ |
| Optimize concentrate crude protein content | ✓ | ✓ | ✓ | ✓ |
| Replace soy cake or meal with low emission alternatives e.g., rape cake or field beans | × | ✓ | ✓ | × |
| Feed agro-alimentary by-products | × | × | × | ✓ |
| Feed additives e.g., lipids, yeast, nitrate, amino acids etc… | × | × | ✓ | ✓ |
|  |  |  |  |  |
| Soil fertility and N fertilizer |  |  |  |  |
| Improve soil pH via liming | ✓ | ✓ | ✓ | × |
| Optimize soil N, P and K levels | ✓ | ✓ | ✓ | ✓ |
| Optimize mineral N fertilizer application via precision technologies e.g., GPS^1^ | × | ✓ | × | ✓ |
| Incorporate legumes into the sward (clover) | ✓ | ✓ | ✓ | × |
| Replace mineral fertilizer with organic | × | ✓ | × | ✓ |
| Change from CAN^2^ to Urea fertilizer | ✓ | ✓ | ✓ | ✓ |
|  |  |  |  |  |
| Management of stored manure |  |  |  |  |
| Extend length of grazing season | ✓ | ✓ | ✓ | × |
| Cover manure store e.g., UV-stabilised plastic covers, peat, straw or wood chips | ✓ | ✓ | ✓ | ✓ |
| Store solid manure on solid impermeable floor equipped with a drainage system | ✓ | ✓ | ✓ | ✓ |
| Anaerobic digestion/biogas | × | ✓ | ✓ | ✓ |
| Aeration | × | × | × | ✓ |
| Composting | × | × | ✓ | × |
| Partial/total replacement of deep litter with fully slatted floor | × | × | ✓ | ✓ |
| Install fans to reduce straw bedding | × | × | × | ✓ |
| Air cleaning systems (e.g., scrubbers) | × | × | ✓ | × |
|  |  |  |  |  |
| Manure treatment |  |  |  |  |
| Nitrification inhibitor | × | ✓ | × | ✓ |
| Urease inhibitor | ✓ | × | × | ✓ |
| Acidification | × | × | × | × |
| Solids separation | × | × | × | × |
| Low emission slurry spreader | ✓ | ✓ | ✓ | ✓ |
| Manure injection (Rapid soil incorporation) | × | ✓ | ✓ | ✓ |
|  |  |  |  |  |
| Energy |  |  |  |  |
| Increase renewable energy use e.g., solar | ✓ | ✓ | ✓ | ✓ |
| Low energy lighting | ✓ | ✓ | ✓ | ✓ |
| Minimize electricity consumption by using metering devices | ✓ | ✓ | ✓ | ✓ |
| Match tractor power to field work task | ✓ | ✓ | ✓ | ✓ |
|  |  |  |  |  |
| Carbon sequestration |  |  |  |  |
| Preserve or increase permanent grasslands | ✓ | ✓ | ✓ | ✓ |
| Maintain or plant hedgerows/trees | ✓ | ✓ | ✓ | ✓ |
| Minimum or no till | ✓ | × | × | ✓ |

^1^ Global positioning system.

^2^ Calcium ammonium nitrate.

References

Batalla I, Gutiérrez-Peña R, del Hierro O, Pérez-Neira D and Mena Y 2014. Estimation of greenhouse gas emissions from cattle and sheep farming in dehesas of Andalusia (Estimación de las emisiones de gases de efecto invernadero de la ganadería bovina y ovina ecológicas en dehesas de Andalucía). In Proceedings of XI SEAE congress Spanish society for organic farming – Organic family farming, 1-4 October 2014, Vitoria-Gasteiz (Alava), Spain, pp. 1-17.

Boselli L 2015. Environmental assessment of the main northern Italy beef production systems using an LCA methodology. PhD thesis, University of Milan, Milan, Italy.

Carbon Trust 2013. Carbon-Footprinting software - Footprint Expert. The Carbon Trust, Dorset House, Stamford Street, London. <http://www.carbontrust.com/software>

Casey JW and Holden NM 2006. Quantification of GHG emissions from sucker-beef production in Ireland. Agricultural Systems 90, 79-98.

Chadwick DR, Pain BF and Brookman SKE 2000. Nitrous Oxide and Methane Emissions following Application of Animal Manures to Grassland. Journal of Environmental Quality 29, 277-287.

Clarke AM, Brennan P and Crosson P 2013. Life-cycle assessment of the intensity of production on the greenhouse gas emissions and economics of grass-based suckler beef production systems. The Journal of Agricultural Science 151, 714-726.

CNMC, 2017. Listado de Informes de Etiquetado de Electricidad. Retrieved on the 02 August 2018 from <https://gdo.cnmc.es/CNE/resumenGdo.do?anio=2017>

Crosson P, Brennan P and O’Kiely P 2013. An industry approach to measuring greenhouse gas emissions from Irish beef cattle production systems. In: Proceedings of the 5^th^ Greenhouse Gases and Animal Agriculture Conference, 24 to 26 June 2013, University College Dublin, Ireland, p 334.

Dollé J-B, Manneville V, Gac A and Charpiot A 2011. Emissions of greenhouse gases and energy consumption of French beef and sheep meat: Bibliographical review of agricultural assessments. Institut de l’Elevage, Service Batiment – Environment, Paris, France.

Dominguez A 1997. Tratado de fertilizacion (Fertilization Treaty). Mundi-Prensa, Madrid, Spain.

Doreau M, van der Werf HMG, Micol D, Dubroeucq H, Agabriel J, Rochette Y and Martin C 2011. Enteric methane production and greenhouse gases balance of diets differing in concentrate in the fattening phase of a beef production system. Journal of Animal Science 89, 2518-2528.

Duffy P, Hanley E, Hyde B, O'Brien P, Ponzi J, Cotter E and Black K 2014. Ireland national inventory report 2012. Greenhouse gas emissions 1990-2012 reported to the United Nations framework convention on climate change. Environmental Protection Agency, Johnstown Castle Estate, Co. Wexford, Ireland.

Duffy P, Hanley E, Barry S, Hyde B and Alam MS 2015. Ireland informative inventory report 2013. Air pollutant emissions in Ireland 1990-2013 reported to the secretariat of the UN/ECE on long range transboundary air pollution. Environmental Protection Agency, Johnstown Castle Estate, Co. Wexford, Ireland.

del Hierro O 2017. Experiences of calculation of sustainability in the agricultural and livestock sector of the Basque country. Basque Ecodesign meeting, Palacio Euskalduna, 19^th^-20 September. Retrieved on the 2 February 2018 from <http://bem2017.basqueecodesigncenter.net/calendario/sesion-paralela-sectorial-5/>

Ecoinvent 2010. Ecoinvent Centre. Ecoinvent 2.0 database. Swiss centre for life cycle inventories, Dübendorf. Retrieved on the July 5, 2019 from: [www.ecoinvent.ch](http://www.ecoinvent.ch).

Emilia Romagna Agriculture and Fishing 2018. LIFE+ Climate changE-R: Final results. Retrieved on the 14 March 2018 from <http://agricoltura.regione.emilia-romagna.it/climatechanger/temi/risultati-finali>

European Council, 1991. Directive 91/676/EEC of 12 December 1991 concerning the protection of waters against pollution caused by nitrates from agricultural sources. Official Journal of the EU L375, 1-8.

Foley PA, Crosson P, Lovett DK, Boland TM, O’Mara FP and Kenny DA 2011. Whole-farm systems modelling of greenhouse gas emissions from pastoral suckler beef cow production systems. Agriculture, Ecosystems & Environment 142, 222-230.

Gac A, Cariolle M, Deltour L, Dollé J-B, Espagnol S, Flénet F, Guingand N, Lagadec S, Le Gall A, Lellahi A, Malaval C, Ponchant P and Tailleur A 2011. GES’TIM – contributions for the environmental assessment of agricultural activities (GES’TIM - des apports pour l’évaluation environnementale des activitiés agricoles). Innovations Agronomiques 17, 83-94.

Howley M, Dennehy E, Holland M and O'Gallachoir B 2011. Energy in Ireland 1990-2010. Sustainable Energy Authority of Ireland, Energy policy statistical support unit, Dublin 2, Ireland.

Hutchings NJ, Jorgenson MS and Vejlin J 2013. Farm-scale modelling of greenhouse gas emissions from livestock production; bridging the gap between dynamic and static modelling. In: Proceedings of the 5th Greenhouse Gases and Animal Agriculture Conference, 24 to 26 June 2013, University College Dublin, Ireland, pp. 291.

Hyde BP, Carton OT, O'Toole P and Misselbrook TH 2003. A new inventory of ammonia emissions from Irish agriculture. Atmospheric Environment 37, 55-62.

IPCC 2006. Intergovernmental Panel on Climate Change guidelines for national greenhouse inventories. Vol. 4. Agriculture, forestry and other land use. (ed. HS Eggleston, L Buendia, K Miwa, T Ngara, and K Tanabe). Institute for Global Environmental Strategies (IGES), Hayama, Japan.

Met Eireann 2013. Climate of Ireland - Air temperature. Retrieved on the 22 September 2018 from <http://www.met.ie/climate-ireland/surface-temperature.asp>

Ministry of Agriculture, Fisheries, Food and the Environment (MAPAMA) 2017. Spanish national inventory of greenhouse gas emissions 1990-2015 (Espana inventario nacional de emisiones de gases de effecto invernadero). Government of Spain, Secretary of State for Environment, Madrid, Spain.

Misselbrook TH, Chadwick DR, Chambers BJ, Smith KA, Williams J, Demmers T, 2007. Inventory of ammonia emissions from UK agriculture 2006: Inventory submission report November 2007. Department for Environment, Food and Rural Affairs, London, United Kingdom.

Murphy B, Crosson P, Kelly AK and Prendiville R 2017. An economic and greenhouse gas emissions evaluation of pasture-based dairy calf-to-beef production systems. Agricultural Systems 154, 124-132.

Oenema O, Velthof GL, Yamulki S and Jarvis SC 1997. Nitrous oxide emissions from grazed grassland. Soil Use and Management 13, 288-295.

Sauvant D and Nozière P 2016. Quantification of the main digestive processes in ruminants: The equations involved in the renewed energy and protein feed evaluation systems. Animal 5, 755-770.

Soussana JF, Tallec T and Blanfort V 2010. Mitigating the greenhouse gas balance of ruminant production systems through carbon sequestration in grasslands. Animal 4, 334-350.

Urbano P 2010. Tratado de Fitotecnia General (Treaty of general plant technology). Mundi-Prensa, Madrid, Spain.

Veysset P, Lherm M, Bébin D, Roulenc M and Benoit M 2014. Variability in greenhouse gas emissions, fossil energy consumption and farm economics in suckler beef production in 59 French farms. Agriculture, Ecosystems & Environment 188, 180-191.

Yan T, Agnew RE, Gordon FJ and Porter MG 2000. Prediction of methane energy output in dairy and beef cattle offered grass silage-based diets. Livestock Production Science 64, 253-263.

Yan T, Frost JP, Keady TWJ, Agnew RE and Mayne CS 2007. Prediction of nitrogen excretion in feces and urine of beef cattle offered diets containing grass silage. Journal of Animal Science 85, 1982-1989.
